# Lectures on Typhoid Fever, Delivered in the Hotel Dieu Hospital of Lyons

**Published:** 1849-02

**Authors:** 


### WESTERN JOURNAL

OF

### MEDICINE AND SURGERY.

FEBRUARY, 1849.

Art. I.—*Lectures on Typhoid Fever, delivered in the Hotel Dieu Hospital of Lyons.* By M. Brachet. Translated by David W. Yandell, M.D., of Louisville, Kentucky.

These Lectures, delivered in the summer and fall of 1847, were published during the succeeding winter and spring in the *Gazette des Hôpitaux*, and excited an unusual amount of attention among the medical men of Paris. I read them as they successively appeared and more than once had taken my pen in hand to translate them, but still put off the task to "a more convenient season." If that season has not yet arrived, the prevalence of typhoid fever in so many districts of our country seems to me to render the translation particularly opportune at the present time. No better monograph on the subject, perhaps, has ever appeared than these lectures of M.

Brachet, and my belief is that it would be difficult to find more valuable matter in the same compass.

Cholera has appeared in New Orleans and New York and seems to be impending the country. If it should become epidemic it is a problem which physicians will observe with interest, how far the character of the disease is modified by the prevailing diathesis. When the epidemic came before, it was in the wake of scarlatina; now it is supervening upon typhoid fever. Will not this, probably, impress some new features in the progress of cholera.

In undertaking this translation, I have had nothing in view but to be useful, and have therefore used some discretion in the omission of passages which do not bear directly upon the subject. These, however, are not numerous, and in no case has any thing been omitted which has practical interest for readers in this country. My aim has not been to present a literal transcript of the author's thoughts—to preserve the peculiarities of his style—but to give a liberal translation of his valuable matter. The lectures will be continued through a series of numbers until all are given.

*Gentlemen:*

Of the nine cases of typhoid fever that you have seen this morning, in five the disease is of a mild, and in four of a violent form; three of the former are females, the remaining six are males. In certain of the patients the sanguine temperament, in others, the sanguineo-lymphatic temperament, prevails; all are young, the oldest not yet being 25 years of age, of strong constitutions, and previous to their present illness have always enjoyed good health. Authors are generally agreed, and the present cases support the opinion, that youth and early manhood, if not the only period in which the disease occurs, offer, at least, a strong predisposition, although it is not the less to be doubted that it sometimes attacks infancy, middle, and old age. Every temperament is alike subject to it, and if it has sometimes appeared to you that the sanguineo-lymphatic temperament is more exposed to it than any other, it is, I imagine, merely because this temperament predominates amongst us. Neither sex is exempt from it. We generally have as much in the male as in the female wards, and if the number is greater in the one to-day, the relative proportion will be changed to-morrow. You must not suppose because it is now summer, that this season exercised any influence in the production of the disease. The coincidence proves only that it may manifest itself at this season, though it by no means establishes that is it confined to this period of the year. Last year, those of you who were here will recollect it was in February, March, and April that it prevailed. In other years it has made its appearance in December and January, and in others again in the autumn. What we have said of the production of the disease is applicable also to atmospherical conditions, as heat, cold, moisture, dryness, etc.

What, then, are the causes of typhoid fever? Of the nine patients to whom we have called your attention, six are unable to assign any cause for their illness. One attributes it to having become chilled after being heated by violent exercise; the remaining two carry it a step farther and accuse the lassitude or prostration consecutive upon the chilliness. In the great majority of cases typhoid fever recognizes no manifest cause. It is developed alone, spontaneously, and without any apparent influence. You may have remarked that many months sometimes elapse without our seeing a single case of the fever, when one is received into our wards, and this is speedily followed by others. This would indicate an influence on the part of the atmosphere; indeed a like coincidence cannot be otherwise explained. What are the qualities of this influence? Or rather, does the air possess qualties to which we may attribute this influence? All that has been said on this subject is erroneous. It is neither season, nor temperture, a hygrometric, nor an electrical condition of the atmosphere that must be charged with the production of typhoid fever, for it appears indiscriminately with or without these conditions. There must, then, be in the atmospere a peculiar and special state or condition which our senses and instruments cannot appreciate or determine, but which is not the less capable of acting upon the human economy. This state or condition is neither constant nor regular in its appearance, since typhoid fever is sometimes months and even years without appearing, and does so at all seasons of the year indifferently. What is this state,—in what does it consist? Here are boundaries that we cannot pass. Beyond the bare fact, all is uncertainty and mere hypothesis. It is a condition of the atmosphere analogous to that which produces all epidemics—we cannot say more than this. The disease arises, rages with fury, and subsides without our being able, by any of the agents that we now possess, to detect the slightest change in the qualities of the atmosphere. Had authors properly weighed this *quid ignotum*, we should have had none of those interminable and fruitless discussions to which the subject has given rise. We may admit that typhoid fever reigns epidemically without its necessarily constituting one of those great epidemics which decimate populations. To constitute an epidemic form—in order to be considered an epidemic—it is sufficient that a certain number of cases arise in the same locality, under the same atmospherical influences, and without any appreciable cause. Although this state of the atmosphere is met with rather in large cities, it is nevertheless also observed in the country. We have seen the disease prevail with great intensity in the most elevated and apparently salubrious places, at considerable distances from any city.

Three of our patients attribute the cause of their disease to being chilled. We may admit this cause also, though it is less frequent than the preceding. It may perhaps, be observed that we cannot attribute to such a cause a disease which may be developed independently of it during a time when it prevails. In the present instance, we admit the doubt, though we do not wish to be understood as doing so absolutely, for we have repeatedly seen typhoid fever declare itself in subjects who were chilled after being heated. We also think that this affection may be developed sporadically under the influence of the general causes of disease, especially in an air vitiated by dense populations, hospitals, dissecting rooms, and sewers. We moreover believe that it may be produced by another disease which having solicited, so to speak, the pathological modification, may be transformed into typhoid fever. We may cite in support of this opinion the case of a girl, who, you will remember, occupied bed No. 8, in whom the most intense enteritis introduced all the symptoms of typhoid fever. We might detail many similar facts, but this is sufficient for our present purpose. We will return to this subject in another lecture.

Pathologists have written much on the contagion and non-contagion of typhoid fever; some admitting contagion, as MM. Bretonneau, Gendron, Ruef, Elliottson, etc. The greater number of authors, however, reject this opinion. Our own individual experience teaches us that it is non-contagious. None of the patients whom we have treated contracted the disease from another; none have given it to another, and our wards never contain any but those who come from without; neither the other patients, the nurses, or persons in attendance, ever contracting it. Things transpire in the same way in the city, outside the walls of the hospital. Yet we have repeatedly seen a number of persons in the same house attacked in quick succession by the disease. Under such circumstances, has the disease been communicated by one individual to another, or has it not rather been produced by a common cause—the air they have breathed? Although, as we have said, our own observation leads us to adopt the doctrine of non-contagion, we would by no means be hasty in pronouncing on the subject, for observation also teaches us that localities, and certain conditions of seasons and temperaments, may exercise an influence upon and impart a character to the disease, in one situation, which it does not possess in another, and that what we see to-day may change by the morrow. Bretonneau, Gendron, etc. are too good observers to reject their opinion. Since they have seen the disease transported from one place to another by individuals laboring under it, and have been able to follow its progress from house to house; since in these cases typhoid fever has been contagious in certain localities, as in Tours, Dublin, Edinburgh, etc., and at certain periods and in other epidemics may become so, in places where it has not yet assumed this form. You should never lose sight of these shades and variations. It is not only in typhoid fever that they manifest themselves: you will repeatedly have occasion to observe them in other diseases.

Typhoid fever presents itself to you with such well-marked and easily understood characters that you will readily recognize it at first sight. It must astonish you then, to learn that there still are pathologists who express doubts as to its existence. This is not the place to engage in the discussion of this question,—we will simply point out to you the phenomena whereby the disease unfolds itself to us. Instead of detailing to you didactically the progress of the various phenomena at the different periods of the disease, we will content ourselves with classifying them in a way that will permit us both to appreciate their value, and, at a later period, to deduce important conclusions relative to the character of the malady. Bear in mind, however, in this connection, that authors have admitted three periods in the course of the affection; the first week constituting, in the great majority of cases, the first period; the second and sometimes the third week, constituting the second period; and finally, the fourth week constituting the third period.

Although the cerebral, digestive, and circulatory apparatus are the seat and agents of the principal and essential phenomena of the disease, other organs nevertheless do not remain strangers to it; all or nearly all participating more or less in the morbid action. The organs of respiration, secretion, etc., take equal part in it. The phenomena may be divided into two orders. The one will comprehend all those that depend upon the cerebro-spinal apparatus, under the influence of which they are executed. The other will embrace all those that proceed from organic life, and which are under the control of the ganglionic nervous system. The cephalalgia which appears at the commencement of the disease; the vitiations and perversions especially of hearing, which often detects noises that do not exist, and which, on the other hand, is frequently paralyzed to a greater or less degree; the vitiations of the other senses, although less frequent and especially less apparent; the diminution and even abolition of sentiment; the obtusion of the intellectual faculties, and, at a more advanced period, their abolition, the delirium, sometimes somnolency, watchfulness, coma, lassitude, or rather the excessive feebleness of the muscles; the slowness of the movements, and the almost complete immobility of the members, which allows the body to slide by its own weight to the base of the pillow or towards the foot of the bed; later, carphologia, subsultus, paralysis of the extremities, bladder, and rectum, are the phenomena that belong to the first order or cerebral nervous system. The variations presented by the general circulation, first on the part of the pulse, which, in the early periods of the disease, is full and quick, then becoming feeble and accelerated in some instances to 150 beats and more in a minute, and finally, intermits; the peculiar state of turgescence of the capillaries, especially in the digestive mucous membrane from the mouth to the anus, where it gives rise to a great variety of phenomena, of redness, tumefaction, diptheritis, aphthæ, papulæ, pustules, patches, and sometimes to hemorrhages, which are always the source of great alarm; the varied redness of the skin, its miliary, lenticular, petechial, sudaminal eruptions; its eschars, etc.; the congestion of certain parenchymatous organs, as the lungs, liver, and spleen, which are sometimes so seriously compromised; the various secretions, especially in the digestive mucous membrane, from which is furnished the pearly white coat to the gums which Ranque has made a pathognomonic sign; to the tongue its white, yellow, brown, fuliginous, dry or moist, uniform or fissured appearance; to the intestines, the mucous secretion which so readily becomes diarrheic; certain sweats, sometimes abundant and peculiar, at other times suppressed, leaving the skin dry, sharp, and burning; the altered secretions of the bile and urine; the disorder of nutrition, which sometimes presents bloating of the tissues previous to emaciation in appearing to dissolve them; finally, the anomalies presented by the respiratory and digestive organs in certain of their functtons, are the not less important and essential phenomena of typhoid fever, which belong to the second order of organs, the ganglionic nervous system.

It is of little advantage, however, to have thus enumerated the phenomena of typhoid fever, since a thousand other diseases present the same or those analogous to them. This is not sufficient to make you acquainted with the disease; it is necessary, in order to accomplish this, to weigh these different phenomena, and discover if possible if there be those which are pathognomonic of the disease in question.

Among the cerebral phenomena the greater part are common to other affections; such are cephalalgia, watchfulness, delirium, hallucinations, the vitiations and diminution of the senses, somnolence, and coma; certain of these, however, possess a peculiar impress and seem to belong exclusively to typhoid fever. Thus, the lassitude in no wise resembles that of fatigue, or that experienced at the onset of other diseases; it is a prostrating lassitude, without pain, without suffering, without torpor; it is a loss of muscular power *(amyotilité;)* without paralysis. The patient has neither the will nor apparently the ability to move, although he is able, and when he wishes can move.

The most characteristic, perhaps, of all the phenomena is the torpor which seizes the intellectual faculties, and more especially the moral sentiments, as also those of affection, amounting very often to complete prostration or entire suspension of them. The patient still possesses all his wonted intelligence, but it is torpid. His mind is sluggish, inactive; entertaining no thought, following no idea. He replies correctly to all questions addressed to him, but he answers slowly and with indifference. He no longer seems interested in anything, not even in his dearest friends. Modesty, especially, appears lost; the most chaste woman no longer manifests any. When we announced the convalescence of the young man lying in bed No. 4, you might have remarked that when we uncovered him in order to examine his abdomen he, through modesty, drew the bedclothes back over his genitals, which was the first time during his sickness that he had done so. This torpor of the intellectual and sentimental faculties gives to the physiognomy, and especially to the eyes, that imbecile and vacant expression which will seldom deceives, and in most instances enables you very readily to recognize the disease.

Some of the ganglionic phenomena, like the preceding, are peculiar to this, while others are common to other diseases. The latter are numerous. Neither the state of the tongue, the diarrhea, pulse, perspiration, nor urine, present anything peculiar to typhoid fever. There have been so many variations in the appearance of the blood, as well as in the results of the microscopic examinations to which it has been submitted, it sometimes presenting characters identical with those of the blood of the most healthy persons, that, although it has occasionally undergone real changes, we will not stop to speak of a subject so barren of substantial proof, and consequently one which may so easily lead us into error.

Dr. Ranque, of Orleans, believes that he has discovered a pathognomonic sign in the pearly white coat which covers the lateral gums. This product of secretion exists, it is true, in all our patients, and we have met with it in almost all the patients whom we have observed; but it also exists in all the inflammatory states of synochus. We have repeatedly shown you this coat, well characterized, in cases in which there was nothing to induce us to suspect the existence of typhoid fever. It would have been a grave error to have treated the disease upon a belief in the truth of this sign.

You are already aware that although sudamina frequently appear upon the neck, chest, arms, and abdomen, they by no means belong exclusively to typhoid fever. We have observed them in all diseases accompanied by copious perspiration, but most frequently in rheumatism, puerperal affections, and mucous fever. They are consequently not a special sign, and are very often even altogether wanting. They rarely supervene before the second week, as you will remark is true of the cases before us.

A remark presents itself here. The name of sudamina given to this pathological appearance is not always appropriate. Some patients scarcely perspire, and they present an intense eruption; others perspire prodigiously without presenting any trace of one. After continuing about a week the eruption generally subsides. Although the patient continues to perspire abundantly, as is the case in the occupant of bed No. 17, no more sudamina, no more eruptions occur. This phenomenon presents, then, something more than an eruption in some degree mechanical. It appears to us far more intimately connected with the march of the disease, than with the existence of the *diaphrase.*

The rose colored patches or lenticular blotches of the abdomen would be a pathognomonic sign, if they were always present. We have never observed them in any disease but this, but unfortunately they are sometimes wanting, and are rarely ever developed during the first week. It is not until the second or even the third week that they are manifest, and if, as very frequently happens, the disease continues longer than this time, they disappear. This phenomenon, however, although in our opinion pathognomonic, is not of much value, since its temporary and fugacious existence does not admit of its being always found. We have repeatedly shown you that these eruptive stains or blotches, although often confounded with petechiæ, have, nevertheless, no resemblance to them. We will not speak of this petechial eruption, because it has not obtained in the patients whom we have seen in the hospital, and I will add that I have never met with it at Lyons in typhoid fever.

Nor will I speak of the meteorism, of the more or less obtuse pain of the abdomen, of the *gargouillement* in the ileo-cœcal region; of the urine with its variable characters; we have seen how little reliance can be placed on these phenomena, as signs in this disease, since they are often entirely wanting, and are found also in various other diseases.

Two questions here arise: must all these phenomena exist in order that typhoid fever be present? do they all manifest themselves simultaneously? No, the presence of all these phenomena is not necessary to characterize the disease. Certain of them have been wanting among some of our patients, that were developed in others with great intensity, without its by any means following from this that the disease was not the same in both instances. The only phenomenon which has appeared to us to be constant, particularly after the first days, is the moral insensibility, with the expression of stupor in the physiognomy of the patient.

